# Neurological and psychological effects of long COVID in a young population: A cross-sectional study

**DOI:** 10.3389/fneur.2022.925144

**Published:** 2022-08-17

**Authors:** Cristiana Alessia Guido, Fabio Lucidi, Fabio Midulla, Anna Maria Zicari, Elena Bove, Federica Avenoso, Ilaria Amedeo, Enrica Mancino, Raffaella Nenna, Giovanna De Castro, Martina Capponi, Bianca Laura Cinicola, Giulia Brindisi, Flavia Grisoni, Manuel Murciano, Alberto Spalice, the Long-Covid Group of Department of Maternal Sciences

**Affiliations:** ^1^Department of Maternal Sciences, Sapienza University of Rome, Rome, Italy; ^2^Department of Developmental and Social Psychology, Sapienza University of Rome, Rome, Italy

**Keywords:** long-COVID syndrome, COVID-19 (coronavirus disease 2019), SARS-CoV-2 (severe acute respiratory syndrome coronavirus 2), CNS (central nervous system), psychological effects, children, adolescents, pediatrics

## Abstract

**Aim:**

We evaluated the long-term clinical status of pediatric patients after testing positive for COVID-19. We hypothesized that there are similar symptoms to those that have been described in adults and children and that pediatric patients with neurophysiologic symptoms still present 3–5 months after infection have psychological consequences that interfere with their adaptive functioning.

**Method:**

We recruited 322 COVID-19-positive pediatric patients, between 1.5 and 17 years old, from the outpatient clinic for COVID-19 follow-up. Neurological symptoms were analyzed at onset, after 1 month, and after 3–5 months. A psychological assessment with standardized questionnaires was also conducted to determine the impact of the disease.

**Results:**

At the onset of COVID-19, 60% of the total sample exhibited symptoms; this decreased after 1 month (20%) but stabilized 3–5 months after disease onset (22%). Prevailing long-COVID neurological symptoms were headache, fatigue, and anosmia. In the 1.5–5-year-old subgroup, internalizing problems emerged in 12% of patients. In the 6–18-year-old subgroup, anxiety and post-traumatic stress showed significant associations with neurological symptoms of long COVID.

**Conclusions:**

These data demonstrate that long COVID presents various broad-spectrum symptoms, including psychological and long-lasting cognitive issues. If not treated, these symptoms could significantly compromise the quality of life of children and adolescents.

## Introduction

Coronavirus disease 2019 (COVID-19), a manifestation of infection with severe acute respiratory syndrome coronavirus 2 (SARS-CoV-2), was declared a pandemic by the World Health Organization (WHO) on March 11, 2020 (1). During the initial stages of the emergency, Italy witnessed a rapid and massive spread of the infection, which initially led the country to be the most affected by the virus, with many cases and a large number of deaths (2).

Researchers have detailed a range of neurological symptoms in SARS-CoV-2-infected patients (2–5). Due to its local or peripheral presence, in adults the virus could lead to systemic inflammatory responses. Moreover, the increase in cytokine levels has been associated with severe neurological dysfunctions (6). These changes are involved in the pathophysiology of various psychiatric disorders, such as depression, anxiety, post-traumatic stress disorder (PTSD), and obsessive-compulsive disorder (7, 8) in somatic symptoms like body pain and respiratory distress (9, 10) and in neurophysiological alterations such as dysregulation of sleep/wake rhythms and nutrition, the presence of fatigue, and increased inattention and irritability (11, 12).

COVID-19 symptoms can be acute or chronic and persist for a long time. For this reason, *long COVID* or *post-COVID syndrome* is defined as symptoms that persist for > 3 months after onset (13–15). A systematic review and meta-analysis found persistent symptoms in the 80% (95% CI 65–92) of adult population. The most frequent were fatigue, headache, attention deficit disorder, hair loss, and dyspnoea (16). Long-term studies in paediatric patients have reported both neurological problems (headache, fatigue, myalgia, and loss of taste or smell), (13, 17–21) cognitive issues (memory, language, and attention), and neuropsychiatric symptoms (anxiety, depression, and PTSD) (9–11, 22, 23). The persistence of long-term neurological symptoms in adults as well as children and adolescents could be explained by hypometabolism-like brain patterns with long COVID, involving the medial temporal bilateral lobes, brainstem, cerebellum, and the olfactory gyrus right after correction of the small volume (24). However, there is limited information on systematic long-COVID results in the paediatric and adolescent population. Some authors highlight the emergence of psychological symptoms and emotional problems in the child and adolescent population due to the consequences of quarantine (25), highlighting the need to enhance mental health services in the community (26).

The main objective of the study was to detect the presence and type of long COVID symptoms, 3–5 months after onset, to determine similarities with those described by previous studies. We hypothesised that there are similar symptoms to those in adults and children described in the scientific literature. We also hypothesised that patients with neurophysiologic symptoms still present 3–5 months after infection have psychological consequences that interfere with their adaptive functioning.

## Methods

This study was conducted between February and November 2021 at the post-COVID outpatient clinic of the maternal-infant ward of the Umberto I University Hospital in Rome, Italy. We included young people between the ages of 1.5 and 17 years who had contracted SARS-CoV-2 and excluded all patients whose parents did not provide informed consent and all subjects who were not present at follow-ups. The cohort consisted of 322 patients aged 1–17 years (167 males, 165 females, mean age = 10 years, range 1.5–17 years) who had recovered from COVID-19. All patients were seen at the onset of the disease and then 1 month and 3–5 months after infection. During the visits, doctors investigated the children's neurophysiologic conditions and completed the COVID-19 symptom checklist. At the last meeting (3–5 months after infection), children and their parents also completed psychological questionnaires to investigate long COVID symptoms. The study included all paediatric patients who came to the clinic for follow-up. They had previously undergone medical examinations at the clinic.

### Statistical analyses

Statistical analysis was conducted by using SPSS 18.0 for Windows (SPSS, Chicago, IL, USA). Frequency analyses and descriptive statistics were performed to describe the sample and symptoms. A correlation matrix (Pearson correlation significant at *p* < 0.01 and *p* < 0.05 [two-tailed]) and the chi-square test (significant at *p* < 0.05) were used to determine the associations between symptoms and the size effect between the variables was measured with the phi coefficient (small effect = 0.1; medium effect = 0.3; large effect = 0.5).

### Materials

#### Description of the tools

A COVID-19 neurophysiologic symptom checklist was created to determine the clinical history of each participant. Doctors completed the list after each visit, indicating the presence/absence of symptoms. Four questionnaires were used for the psychological assessment. The details of each are presented below.

*The Multidimensional Anxiety Scale for Children-2 Self Report* (MASC 2-SR) (27) is a self-administered questionnaire of 50 items for subjects between the ages of 8 and 18. The tool comprises six scales, which measure the main dimensions of anxiety and a general one that indicates the severity and pervasiveness of the symptoms of anxiety. Standardised T scores have a mean of 50 and a standard deviation of 10. T scores > 65 (1.5 standard deviations [SD]) are considered to be of clinical relevance, between 55 and 64 above average, and <54 average.

The *Children's Depression Inventory* (CDI-2 SR) (28) is a questionnaire on depressive symptoms for individuals aged 7–17 years. The tool provides a total symptom score and specific scores divided into two categories: emotional problems and functional problems. T scores > 65 (1.5 SD) are considered to be of clinical relevance, between 60 and 64 above average, and <59 average.

The *Trauma Symptom Checklist for Children-A* (TSCC-A) (29) is a self-assessment questionnaire for children aged 8–16 years, consisting of 44 items and five clinical scales (Anxiety, Depression, Anger, Post-traumatic Stress, and Dissociation). Standardised scores > 65 are considered significant; scores <65 are not significant.

The *Child Behavior Checklist* (CBCL) (30, 31) is a proxy-report questionnaire for parents to report on their children's behaviour. We used two versions: one for individuals aged 1.5–5 years and one for individuals aged 6–18 years. The profile that emerges from the questionnaires consists of a total scale, a scale of internalising problems, a scale of externalising problems, syndromic scales, and scales oriented to the *Diagnostic and Statistical Manual of Mental Disorders*. The 6–18-year-old version also provides a measure of the children's skills (activity, sociability, and school). For both versions of the CBCL, syndromic scale T scores <65 are normal, between 65 and 70 are borderline, and >70 are clinical. For the skill scale in the 6–18-year-old version, T scores <30 are average, between 30 and 35 are borderline, and > 35 are clinical. Scores in the borderline and clinical ranges (> 65) were considered to be alterations from a condition of normality (**Figures 2**, **3**).

## Results

### Sample

The total sample comprised 322 patients aged between 1.5 and 17 years (mean [M] age = 9.53, SD = 3.73, mode = 10 years), of which 50 were preschool age (age group 1.5–5 years; M = 3.5, SD = 1.39 years) and 272 were school age (age group 6–17 years; M = 10.65, SD = 2.83 years). There were 167 boys and 155 girls. Parents who completed the CBCL (56 fathers and 266 mothers) had a mean age of 43 years (range 26–63 years).

### COVID-related neurophysiological symptoms in paediatric patients

At the onset of COVID-19, symptoms occurred in 192 children (60% of the total sample). The remaining 40% of the sample that had no symptoms at onset was recruited only after finding positivity with the swab. After 1 month, 66 patients (20%) showed symptoms, and this level stabilised 3–5 months after the onset of the disease, with 70 patients (22%) showing symptoms.

In the total sample, the prevailing symptom at onset was headache (33.5%), which reduced in frequency and remained after 3–5 months in 7.5% of the sample. Fatigue (27.3% of patients at onset and 6.8% after 3–5 months) and anosmia (19.9% at onset and 2.2% after 3–5 months) were also prevalent. Ageusia followed a non-linear trend: 18% of patients showed this symptom at onset, followed by a reduction after 1 month (3.1%), and an increase after 3–5 months (6.5%). Based on these data, we hypothesised that the loss of taste also occurs more than 1 month after the onset of COVID-19 symptoms. Other symptoms such as myalgia, dizziness, dysgeusia, and ocular pain tended to regress over time, while chest and musculoskeletal pain were not very prominent at any time (Table 1).

**Table 1:** Symptom course in COVID-19-positive paediatric patients.

**Total sample (*****n =*** **322)**						
**Symptoms**	**Onset**		**1 month**		**3–5 months**	
	***N***	***%***	***N***	**%**	***N***	**%**
Headache	108	33.5	18	5.6	24	7.5
Fatigue	88	27.3	37	11.5	22	6.8
Anosmia	64	19.9	14	4.3	7	2.2
Ageusia	58	18	10	3.1	21	6.5
Myalgia	28	8.7	4	1.2	0	0
Muscoloskeletal pain	10	3.1	0	0	2	0.6
Dysgeusia	6	1.9	2	0.6	0	0
Dizziness	5	1.6	0	0	0	0
Chest pain	1	0.3	10	0.3	2	0.6
Eye pain	1	0.3	0	0	0	0

**Figure 1:**
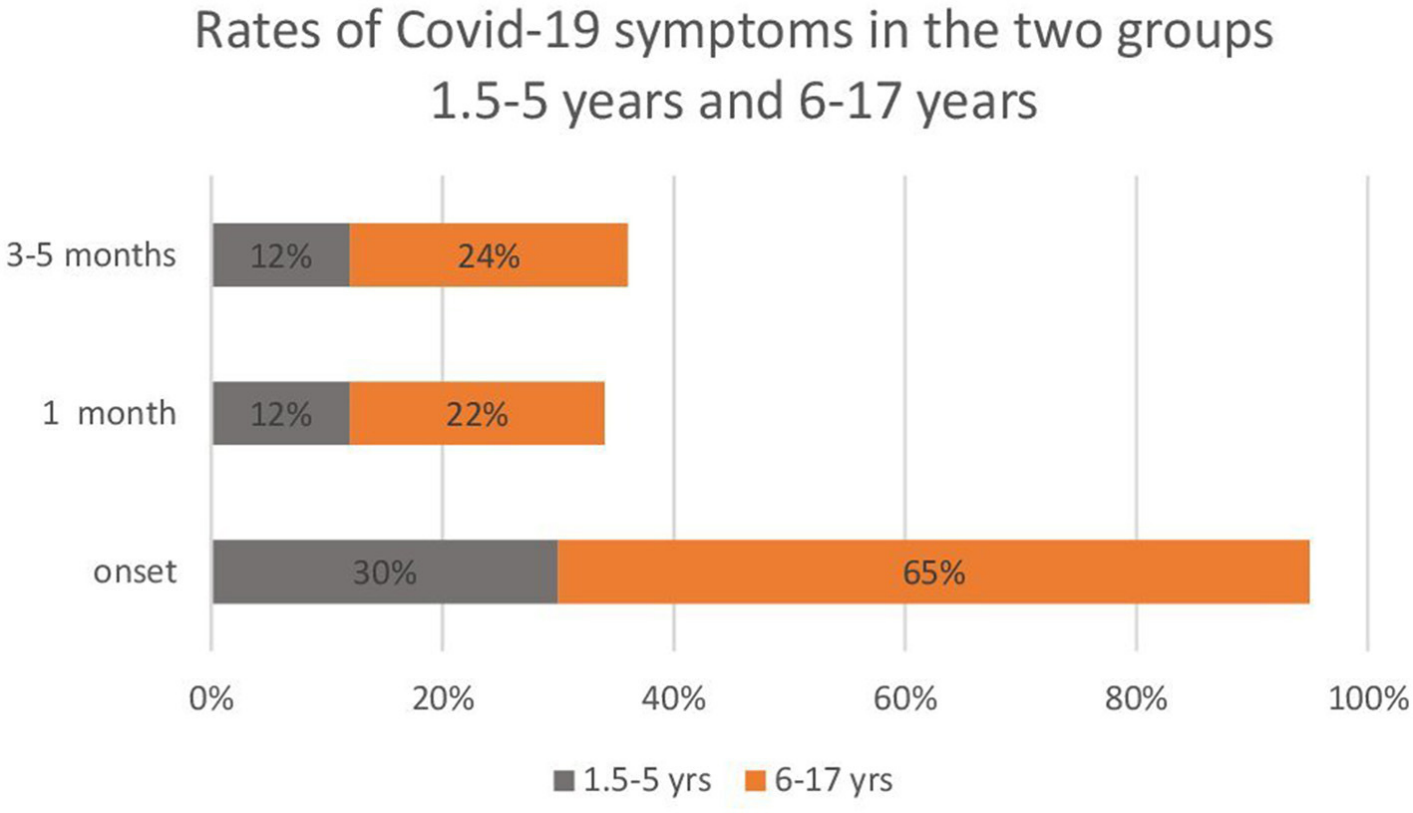
Rates of COVID-19 symptoms in the two subgroups of paediatric patients: 1.5–5 years (*n* = 50) and 6–17 years (*n* = 272).

From the analysis of the partial frequencies carried out separately in the two groups (1.5–5 and 6–17 years), the 1.5–5-year-old patients had fewer symptoms than the 6–17-year-old patients. Moreover, symptoms tended to stabilise in fewer pre-school children compared with school-age children (Figure 1).

### Long-term COVID symptoms

In the total sample, 3–5 months after onset of symptoms, neurophysiological symptoms were detected more frequently in the 6–17-year-old patients (Table 2). The descriptive table summarises the symptoms present in the long term in the two groups (1.5–5 and 6–17 years). Headache (*n* = 23, 7%), fatigue (*n* = 21, 6%), and ageusia (*n* = 21, 6%) were more common in children aged 6–17 years. By constrast, in the 1.5–5-year-old group, headache, fatigue, and ageusia were each present in only 1 patient (0.3%). The other evaluated symptoms only occurred in the children aged 6–17 years: anosmia (*n* = 7, 2%), chest pain (*n* = 2, 0.6%), and musculoskeletal pain (*n* = 2, 0.6%).

**Table 2:** Descriptions and percentages of COVID-19 symptoms in the two subgroups 3–5 months after COVID-19 infection.

**Presence of smptoms at 3–5 months**	**1.5 yrs**		**6–17 yrs**	
	***N***	**%**	***N***	**%**
Headache	1	0.3	23	7
Fatigue	1	0.3	21	6
Ageusia	1	0.3	20	6
Anosmia	0	0	7	2
Chest pain	0	0	2	0.6
Musculoskeletal pain	2	0.6	0	0
Dysgeusia	0	0	0	0
Eye pain	0	0	0	0
Dizziness	0	0	0	0
Myalgia	0	0	0	0

As detailed in Table 3, in the total sample 34 patients (10%) showed changes in eating habits; 41 patients (13%) presented sleep disturbances; 46 patients (14%) had cognitive, behavioural, and mood problems; and 93 patients (29%) had increased their use of technological tools, with a mean increase of 3 h/day (range 1–5 h).

**Table 3:** Changes in symptoms in the two subgroups 3–5 months after COVID-19 infection.

	**Total sample**		**6–17 yrs**		**1.5–5 yrs**	
	***N =* 322**		***N =* 272**		***N =* 50**	
	**N**	**%**	**N**	**%**	**N**	**%**
**Sleep problems**	42	13	37	11.2	5	1.5
**Eating changes**	34	10.6	33	10.2	1	0.3
Food reduction	21	6.6	20	6.2	1	0.3
Food increase	13	4.1	13	4.0	0	0.0
**Behavioral-cognitive**	46	14.3	43	13.3	3	0.9
Attention	29	9.0	28	8.7	1	0.3
Academic impairment	5	1.6	5	1.6	0	0
Irritability	5	16.6	4	1.2	1	0.3
Anxiety	3	0.9	3	0.9	0	0
Auditory hallucinations	1	0.3	1	0.3	0	0
Hyperactivity	1	0.3	1	0.3	0	0
Memory	1	0.3	1	0.3	0	0
Obsessions-compulsions	1	0.3	0	0	1	0.3
**Technologies**	93	28.9	90	27.9	3	0.9

Considering only the 6–18-year-old group, 36 patients (11%) reported problems falling asleep or waking up at night, and 33 patients (10%) reported dietary changes as an increase (*n* = 13, 4%) or a decrease (*n* = 20, 6%) in food intake. In this group, 43 patients (13%) presented cognitive and behavioural problems, in particular attention disorders (*n* = 28, 9%). Moreover, 43 patients (28%) showed an increase in the use of technological tools (such as smartphones, tablets, and PCs). In the 1.5–5-year-old group, 5 patients (1%) reported sleep problems, 1 patient (0.3%) reported eating problems, 3 patients (0.9%) showed cognitive or behavioural problems, and 3 children (0.9%) presented an increase in the use of technology.

### Long COVID psychological conditions

#### Clinical symptoms and adaptive behaviour

**Figure 2:**
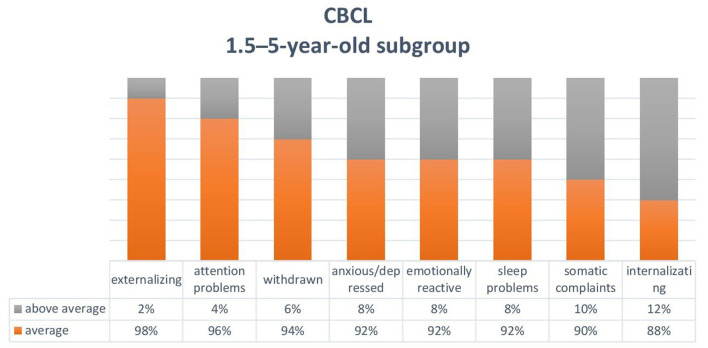
Child Behavior Checklist (CBCL) for the 1.5-5-year-old subgroup.

Based on the CBCL completed by the parents on the behaviour of 50 children aged between 1.5 and 5 years (mean age = 3.4; boys = 30 and girls = 20), some patients had above average scores (T scores > 65) for emotional reactivity (*n* = 4, 8%), anxiety/depression (*n* = 4, 8%), somatic disorders (*n* = 5, 10%), closure (*n* = 3, 6%), sleep problems (*n* = 4, 8%), and attention problems (*n* = 2, 4%). Internalisation problems (*n* = 6, 12%) prevailed over externalisation problems (*n* = 1, 2%) (Figure 2).

**Figure 3:**
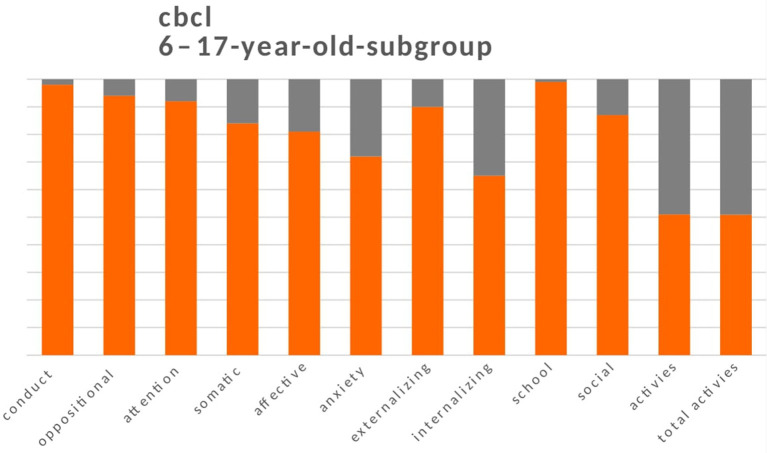
Child Behavior Checklist (CBCL) for the 6-17-year-old subgroup.

For the 272 children aged 6–17 years (mean age = 10.6 years; 137 boys and 135 girls), the parents reported a decline in overall activity in 147 (54%) children (T scores > 30). There was impairment in recreational activities (sports, games, etc.) (*n* = 134, 49%), in the social area (*n* = 36, 13%), and in academic performance (*n* = 2, 1%). Similarly to the 1.5–5-year-old children, more children had internalising problems with borderline or clinical scores (*n* = 96, 35%) than externalising problems (*n* = 8, 10%). Anxiety problems were the most frequent (*n* = 74, 28%), followed by mood problems (closure and depression; *n* = 51, 19%), somatic problems (*n* = 43, 16%), attention problems (*n* = 20, 8%), oppositional problems (*n* = 16, 6%), and conducted (*n* = 6, 2%) (Figure 3).

Figures 2, 3 show the sum of the percentages relating to borderline and clinical scores in the two groups of children aged between 1.5 and 5 years and 6-17 years.

#### Anxious-depressive symptoms

The 6–17-year-old age group was divided into a subgroup of 226 children between the ages of 8 and 16 (mean age = 11.2 years; 117 boys and 119 girls). Some patients showed above average MASC-2 scores (> 55 points) on the social anxiety scale (*n* = 52, 37%) and particularly the performance anxiety subscale (*n* = 90, 40%), followed by feelings of humiliation and rejection (*n* = 57, 25%). There was also separation anxiety in 88 patients (37%), generalised anxiety in 65 patients (28%), obsessive-compulsive symptoms in 52 patients (23%), and fear of danger in 47 patients (21%). There were also high scores in the physical dimension (*n* = 65; 28%) with symptoms of panic (*n* = 71; 32%) and tension (*n* = 67; 30%), as responses to the anxious condition.

The CDI-2 revealed the presence of emotional problems in 61 patients (24%), concerning both negative mood–physical symptoms (*n* = 39, 16%) and negative self-esteem (*n* = 22, 9%). There were functional problems with feelings of ineffectiveness in 33 patients (15%) and interpersonal problems in 27 patients (12%).

We compared anxiety scores with the presence or absence of COVID symptoms 3–5 months after infection by using the chi-square test (Table 4). There were significant associations between long COVID symptoms and subtypes of generalised and social anxiety, obsessive-compulsive symptoms, and physical symptoms.

**Table 4:** Comparison between patients who had symptoms at 3–5 months after COVID-19 infection and those who did not.

**Anxiety subscale MASC2**	*Total	**Present	***Absent	****P-value	*****Effect size
**Group 8–16 years (*****n** **=*** **226)**					
Separation anxiety	83	25	58	0.259	0.070
Generalized anxiety	65	28	37	**0.000**	**0.246**
Social anxiety	82	31	51	**0.003**	**0.201**
Humiliation	57	24	33	**0.001**	**0.212**
Performance anxiety	90	29	61	0.089	0.113
Obsessions-Compulsions	52	23	29	**0.001**	**0.266**
Physical symptoms	65	29	36	**0.000**	**0.268**
Panic	71	30	41	**0.000**	**0.249**
Tension-Restlessness	67	29	38	**0.000**	**0.254**
Avoidance of danger	47	18	29	0,32	0.142

*number of anxiety symptoms in the 8 to 16 year old patient group.

^**^number of patients with long COVID neurophysiological symptoms (>3 months) and anxiety symptoms.

^***^number of patients with anxiety symptoms without long covid neurophysiological symptoms.

^****^Pearson's Chi-Square Test: significance = p-value < 0.05, indicates the significance between long COVID symptoms and anxiety symptoms.

^*****^Phi value = 0.1: small effect, = 0.3: medium effect = 0.5: large effect.

Bold value shows the significant relationships between anxiety symptoms (generalized anxiety, social anxiety, humiliation, etc.) and the presence of 3-month neurological symptoms (statistically significant p-value) and the effect size of significant scores.

#### Post-traumatic symptoms

Post-traumatic symptoms were studied with the TSCC-A self-assessment questionnaire in the subgroup of 226 patients aged 8–16 years. Before filling out the questionnaire, doctors asked the parents to report whether their children had been exposed to traumatic events immediately before, during, or after COVID-19 infection. Only six children had been exposed to traumatic events (*n* = 3, hospitalisation of the mother; *n* = 2, death of the grandfather or uncle: *n* = 1, isolation of positive parents).

Twenty-eight patients (12%) scored high on the Post-traumatic Stress scale, 22 patients (10%) had dissociative symptoms (dissociation index), 26 patients had overt dissociation (11%) and 14 patients showed hidden dissociation (6%). Emotions of anxiety (*n* = 22, 10%), depression (*n* = 23, 10%), and anger (*n* = 19, 8%) also emerged, linked to the traumatic state.

Statistical analysis revealed significant correlations between the presence of long COVID symptoms and the clinical questionnaire scores on the PTSD, Depression, Anger, and Dissociation scales. On the contrary, there were no significant correlations between the traumatic events reported by the parents and the questionnaire scales (Table 5).

**Table 5:** Correlations between post-traumatic stress symptoms (Trauma Symptom Checklist for Children-Adolescent [TSCC-A] scales), stressful events, and the presence of neurophysiological symptoms from COVID-190.

	**Symptoms onset after covid**	**Symptoms 1 month**	**Symptoms 3–5 months**	**Stressfull events**	**Anxiety**	**Depression**	**Anger**	**PTSD**	**Dissociation index**	**Overt** **dissociation**	**Covert dissociation**
Symptoms onset after covid	—										
Symptoms 1 month	00.350**	—									
Symptoms 3– 5 months	00.280**	00.579**	—								
Stressfull events	−00.012	00.099	00.027	—							
Anxiety	00.085	00.092	00.111	−00.054	—						
Depression	00.029	00.048	00.200**	−00.056	00.433**	—					
Anger	−00.079	00.014	00.147*	00.049	00.385**	00.531**	—				
PTSD	00.100	00.100	00.174**	−00.062	00.783**	00.673**	00.515**	—			
Dissociation index	00.085	00.022	00.145*	−00.054	00.547**	00.679**	00.492**	00.601**	—		
Overt dissociation	00.055	00.022	00.070	−00.060	00.537**	00.612**	00.440**	00.580**	00.864**	—	
Covert dissociation	−00.072	−00.060	00.014	−00.042	00.287**	00.217**	00.253**	00.293**	00.287**	00.195**	—

**Correlation is significant at the 000.01 level (2–tailed).

^*^Correlation is significant at the 000.05 level (2–tailed).

Bold value shows significant correlations between PTSD symptoms (symptoms of anxiety, depression and post-traumatic) and neurophysiological symptoms after 3 months of infection.

## Discussion

Our findings highlight many common elements regarding long COVID neurological symptoms with other published studies. First, there is great heterogeneity of long COVID neurological symptoms (18, 19, 21, 22). In our paediatric sample, there were 1*0* initial symptoms, which reduced to six symptoms 3–5 months after the infection. Furthermore, the recurrent symptoms of our sample, such as fatigue, headache, cognitive, and mood problems, were the most frequent in similar studies (18, 19, 23). Neurological sequelae are similar in adults and children (16, 17), and this and this could be explained by brain patterns similar to long COVID hypometabolism, involving the same areas of the brain (5, 24). However, further studies are needed to confirm this hypothesis. Interestingly, we found a non-linear course for the loss of taste (ageusia), which occurred more frequently 3–5 months after the infection (onset = 58; 1 month = 10; 3–5 months = 21). These data suggest that in some subjects, ageusia might appear later than other symptoms. Unlike other published studies, dividing the sample into two age groups allowed us to highlight a lower onset of neurological symptoms and a more significant reduction over time in the group of younger children (1.5–5 years) compared with older children (6–17 years) (Figure 1). The most relevant aspect of our research concerns psychological problems that, as described by other authors, compromise the daily activities in school-age children (19). The standardised scales we used for the assessment (27–29, 31–35) revealed that a large percentage of children aged 8–16 years developed symptoms of social and separation anxiety (37%), panic (32%), tension (30%), obsessive-compulsive tendencies (23%), and generalised anxiety (28%) related to the presence of neurological symptoms (Table 4). Although small in number, some children had depression and PTSD symptoms. These data demonstrate that long COVID syndrome is not limited to neurological problems but presents various broad-spectrum symptoms that include psychological and long-lasting cognitive aspects. If not treated, these aspects could significantly compromise the quality of life of children and adolescents.

## Conclusion

We have found that the most frequent symptoms found in our paediatric sample were similar to those described in the literature and occurred more in the older patients (6–17 years). While neurological symptoms tended to decrease over time, psychological ones were present in greater numbers in 6–17-year-old patients. Recent studies have found similar brain patterns between adult and paediatric COVID-19 populations, but additional longitudinal studies should be conducted in larger cohorts to determine the relationships between COVID-19 infection and the persistence, type, and severity of neuropsychiatric symptoms in different age groups. The results could be useful for planning prevention actions aimed at reducing the risk of chronic symptoms.

### Limitations

Due to time limitations we did not compare symptoms with a control group. Furthermore, we have not found studies similar to ours using standardized psychological scales on patients with Long-Covid, and this aspect does not allow us to make a systematic comparison of symptoms with clinical samples similar to ours. Thanks to the use of standardized tools, the study can be replicated.

## Data availability statement

The original contributions presented in the study are included in the article/supplementary material, further inquiries can be directed to the corresponding author/s.

## Ethics statement

Ethical review and approval was not required for the study on human participants in accordance with the local legislation and institutional requirements. Written informed consent to participate in this study was provided by the participants' legal guardian/next of kin.

## Author contributions

CAG conceptualized the study, selected the psychological data collection tools, performed the data analyzes, and drafted the initial manuscript. EB conceptualized the study, selected tools for collecting neurophysiological data, collected the data, and reviewed the manuscript. MM contributed to the conception and design, analysis and interpretation of the data, and drafting of the article. FM, EM, and RN conceptualized and designed the study, planned the inclusion criteria for study participants, and reviewed and revised the manuscript. AS, FL, and AMZ conceptualized and designed the study, supervised the data collection, and critically examined the manuscript for important intellectual content. IA, FA, and FG designed tools for data collection, collected and interpreted the data, and contributed to the drafting of the article. BLC, GB, MC, and GDC made substantial contributions both to the acquisition of the data and to the drafting of the paper. All authors listed have made a substantial, direct, and intellectual contribution to the work and approved it for publication.

## Conflict of interest

The authors declare that the research was conducted in the absence of any commercial or financial relationships that could be construed as a potential conflict of interest.

## Publisher's note

All claims expressed in this article are solely those of the authors and do not necessarily represent those of their affiliated organizations, or those of the publisher, the editors and the reviewers. Any product that may be evaluated in this article, or claim that may be made by its manufacturer, is not guaranteed or endorsed by the publisher.

## Abbreviations

CBCL: Child Behavior Checklist
CDI-2 SR: Children's Depression Inventory
CNS: Central Nervous System
COVID-19: Coronavirus Disease 2019
M: mean
MASC 2-SR: Multidimensional Anxiety Scale for Children-2 Self Report
PTSD: Post-Traumatic Stress Disorder
SD: Standard Deviation
SARS-CoV-2: Severe Acute Respiratory Syndrome Coronavirus 2
TSCC-A: Trauma Symptom Checklist for Children-Adolescent.
